# Rapid evolution of silver nanoparticle resistance in *Escherichia coli*

**DOI:** 10.3389/fgene.2015.00042

**Published:** 2015-02-17

**Authors:** Joseph L. Graves, Mehrdad Tajkarimi, Quincy Cunningham, Adero Campbell, Herve Nonga, Scott H. Harrison, Jeffrey E. Barrick

**Affiliations:** ^1^Department of Nanoengineering, Joint School for Nanoscience and Nanoengineering, North Carolina Agricultural and Technical State University/University of North CarolinaGreensboro, NC, USA; ^2^Department of Nanoscience, Joint School for Nanoscience and Nanoengineering, North Carolina Agricultural and Technical State University/University of North CarolinaGreensboro, NC, USA; ^3^Department of Biology, North Carolina Agricultural and Technical State UniversityGreensboro, NC, USA; ^4^Department of Biology, Bennett CollegeGreensboro, NC, USA; ^5^Student Research Opportunity Program, Michigan State UniversityEast Lansing, MI, USA; ^6^Department of Molecular Biosciences, The University of Texas at AustinAustin, TX, USA

**Keywords:** eNPs, AgNPs, *E. coli*, Genomics, adaptation

## Abstract

The recent exponential increase in the use of engineered nanoparticles (eNPs) means both greater intentional and unintentional exposure of eNPs to microbes. Intentional use includes the use of eNPs as biocides. Unintentional exposure results from the fact that eNPs are included in a variety of commercial products (paints, sunscreens, cosmetics). Many of these eNPs are composed of heavy metals or metal oxides such as silver, gold, zinc, titanium dioxide, and zinc oxide. It is thought that since metallic/metallic oxide NPs impact so many aspects of bacterial physiology that it will difficult for bacteria to evolve resistance to them. This study utilized laboratory experimental evolution to evolve silver nanoparticle (AgNP) resistance in the bacterium *Escherichia coli* (K-12 MG1655), a bacterium that does not harbor any known silver resistance elements. After 225 generations of exposure to the AgNP environment, the treatment populations demonstrated greater fitness vs. control strains as measured by optical density (OD) and colony forming units (CFU) in the presence of varying concentrations of 10 nm citrate-coated silver nanoparticles (AgNP) or silver nitrate (AgNO_3_). Genomic analysis shows that changes associated with AgNP resistance were already accumulating within the treatment populations by generation 100, and by generation 200 three mutations had swept to high frequency in the AgNP resistance stocks. This study indicates that despite previous claims to the contrary bacteria can easily evolve resistance to AgNPs, and this occurs by relatively simple genomic changes. These results indicate that care should be taken with regards to the use of eNPs as biocides as well as with regards to unintentional exposure of microbial communities to eNPs in waste products.

## Introduction

Experimental evolution is routinely used to study the causes and consequences of natural selection. Specifically experimental evolution can predict how phenotypes and their underlying genomic architecture change in response to new environments. This approach may be particular fertile for bacterial evolution. While these organisms have persisted and diversified for billions of years, they often must respond to novel environments. For example, the exponential increase in products utilizing nanomaterials (NMs) will mean increased exposure to NMs for a variety of microbes. Increasing amounts of metallic/metallic oxide nanoparticles are being used in consumer products. Nano-titanium oxide (nano-TiO_2_) is produced on a large scale for applications in paints, cosmetics, sunscreens, photo-catalysts and solar cells, as well as water purification devices. The predicted concentration of nanoTiO_2_ in European waters for 2009 was 20 ng/L (Gottschalk et al., 2009). Similarly values for nano-silver were calculated at 6.6 μg/kg/year, 526 μg/kg/year, 0.088 μg/L/year, 16.40 μg/L/year, 1.29 mg/kg/year, and 153 μg/kg/year for American soil, sludge, surface water, Sewage Treatment Plant (STP) effluent, STP sludge, and sediment respectively.

In addition, metallic and metallic oxide nanoparticles are being hailed by some as a powerful new weapon against multi-drug resistant bacteria (Rai et al., 2012). This is motivated by recognizing that noble metals such as silver have a long history as antimicrobial agents with use dating back at least to 1000 BCE. In modern times, nano silver, copper, and silica have been successfully used in a variety of settings as both antimicrobial and anti-insecticidal compounds. Nanosilver is now being widely used in food packaging materials and also being proposed for use in surgical gowns and surgical gauze (Li et al., 2006; Siqueira et al., 2014). Silver nanoparticles (AgNPs) have been shown to be protective agents against numerous species of bacteria, including *Escherichia coli*, *Enterococcus faecalis*, *Staphylococcus aureus*, and several others. Other metallic and metallic oxide nanoparticles have also been employed against bacteria including titanium oxide (TiO_2_), magnesium oxide (MgO), copper (Cu), copper oxide (CuO), zinc oxide (ZnO), cadmium selenium (CdSe) and cadmium telluride (CdTe), (Duncan, 2011).

The effectiveness of NPs against both bacteria and viruses are due to their high surface-to-volume ratio and their unique chemical and physical properties. Numerous studies have now shown that the toxicity of NPs against bacteria appears dependent on particle composition, shape, size, and concentration (for AgNPs, concentrations of >75 μg/ml usually completely inhibits growth; Rai et al., 2012; Tajkarimi et al., 2014). While the exact mechanisms of silver nanoparticle toxicity to bacteria are not fully known, there is a growing consensus concerning the candidate actions. First, the action of silver nanoparticles occurs both by the release of silver ion (Ag^+^) as well as from potential disruption or damage to the cell wall and membrane caused by the particles themselves (Rai et al., 2009, 2012; Mijnendonckx et al., 2013). Silver interacts with the thiol groups in respiratory enzymes and other proteins of bacterial cells causing them to become inactivated (Liau et al., 1997; Feng et al., 2000). It also binds to the cell wall and cell membrane inhibiting the respiration process (Klasen, 2000; Rai et al., 2009). Silver is known to act on *E. coli* by inhibiting the uptake of phosphorous and causing the release of phosphate, mannitol, succinate, proline, and glutamine from the cells (Yamanaka et al., 2005; Rai et al., 2012). The penetration of silver ions inside the cell is thought to impact the ability of DNA to replicate by causing it to condense. Thus once inside, Ag^+^ ions may be lethal as they disrupt metabolism, cell signaling, DNA replication, transcription, translation, and cell division, either directly or through the generation of reactive oxygen species (ROS) (Rai et al., 2012; Mijnendonckx et al., 2013). It is the fact that metallic/metallic oxides impact so many aspects of bacterial physiology and reproduction that has led some researchers to suggest that it will be difficult for bacteria to evolve resistance to them (e.g., see discussion in Rai et al., 2012). However, despite this claim, silver-resistant bacteria have been repeatedly found in burn wards, clinical and natural environments, and on human teeth (Mijnendonckx et al., 2013). Given the projections concerning deployment of metallic, particularly silver nanoparticles for bacterial control it is important to evaluate the evolvability of resistance to them (Gupta and Silver, 1998; Graves, 2014). This experiment utilizes a relatively naïve laboratory bacterium, *E. coli* K-12 MG1655, to determine both how quickly resistance to AgNPs can evolve and also to evaluate the nature of the genomic changes responsible for such resistance.

## Materials and methods

### Bacteria

*E. coli* K-12 MG1655 (ATCC #47076) was chosen for this study due to the paucity of known silver or antibiotic resistant loci in this bacterium. There are no plasmids in this strain, and the circular chromosome is composed of 4,641,652 nucleotides (GenBank: NC_000913.3; Riley et al., 2006). Table 1 lists major antimicrobial resistance genes found in various microorganisms, and their presence or absence in MG1655.

**Table 1:** **Major antimicrobial resistance genes in *Escherichia coli***.

**Antimicrobial agent**	**Genes**	**MG1655**	**References**
Streptomycin	*aadA1*	No	Momtaz et al., 2012
Gentamicin	*aac(3)-IIV*	No	
Sulfonamide	*sul1, bla SHV, bla, CMY*	No	
Beta lactams	*kpc, cmy-2, shv, tem*	No	Xia et al., 2011
	*ctx-m, lap-1, bla-tem*	No	
Ampicillin	*ere(A)*	No	
Erythromycin	*cat A1*	No	Momtaz et al., 2012
Chloramphenicol	*cm1A*	No	
Tetracycline	*tet(A), tet(B)*	No	
Trimethoprim	*dfr A1*	No	
Quinolones	*qnr A*	No	
Sulfonamide	*sul1, sul2*	No	Tadesse et al., 2012
Silver	*silE, sil-CFBA*	No	Gupta et al., 2001; Silver, 2003
Silver	*cusS, ompA, ompB, ompF, ybdE*	Yes	Li et al., 1997; Franke et al., 2001; Gudipathy and McEvoy, 2014

### Evolution experiment

We cultured *E. coli* K-12 MG1655 using Davis Minimal Broth (DMB, Difco™Sparks, MD) with Dextrose 10% (Dextrose, Fisher Scientific, Fair Lawn, NJ) as a sole carbon source, enriched with thiamine hydrochloride 0.1% in 10 ml of total culture volume maintained in 50 ml Erlenmeyer flasks. The flasks were placed in a shaking incubator with temperature maintained at 37°C for 24 h. The stock culture was propagated by daily transfers of 0.1 ml of each culture into 9.9 ml of DMB for 11 days of regrowth before selection for AgNP resistance began. The controls were founded by taking five different 0.1 ml samples and adding them to 9.9 ml of DMB broth. These cultures were allowed to grow for 24 h, typically representing 6.5 generations of population growth from ~10^6^ cells per ml at hour 0–10^8^ cells per ml at 24 h. On the following day the treatment groups were founded by taking 0.1 mL samples from each of the controls and adding them to 9.9 ml of standard DMB medium with the addition of 50 μg/L or 50,000 ng/L concentration of 10 nm citrate-coated spherical AgNPs. The nanoparticles were obtained from Nanocomposix, San Diego, CA. The AgNPs were citrate-coated due to the need to prevent agglomeration of the particles. Citrate was chosen for this experiment due to the inability of citrate to be metabolized by *E. coli* (Scheutz and Strockbine, 2005).

Our laboratory has studied the impact of two commonly used silver nanoparticle coating types (citrate and polyvinylpyrolidone, PVP) on bacteria (Tajkarimi et al., 2014). Generally, we have found that citrate-coated AgNPs were more effective against *E. coli* K-12 MG1655 than PVP-coated AgNPs. The impact of citrate coating is most likely due to the charge of citrate impacting the rate of release of Ag^+^ ion, as opposed to the compound itself (El Badawy et al., 2011; Xiu et al., 2012). Control populations were designed (C_1_—C_5_) and those cultured in AgNPs were designed as (T_1_—T_5_). Each five generations, after transfer to found the next generation, the remainder of each replicate was frozen at −80°C for future analysis. The generation counts used in this study are based upon the number of cell doublings of *E. coli* required to grow to saturation each day after dilution in the standard DMB broth (controls grew ~6.5 generations/per day.) We know that the treatment populations were at least 6 generations behind the controls, since they were founded 24 h later. In addition, it is unclear whether the bacteria exposed to AgNPs were able to maintain the same number of cell doublings as the controls due to the presence of AgNPs in their environment. This was most likely the case early in the selection experiment, but later measurements of the treatment populations in the presence of AgNPs suggest that they achieved full growth each day after they acquired adaptations allowing them to persist in the AgNP environment.

It was determined that 50 μg/L (50,000 ng/L) of AgNPs allowed some bacterial growth as indicated by observable turbidity after 24 h of exposure. This concentration was chosen as the starting point for the resistance studies. After 50 generations of culture, the culture concentration of the treatment populations was increased to 100 μg/L (100,000 ng/L). This level was maintained for another 90 generations. After this the selection concentration was increased to125 μg/L (125,000 ng/L). This concentration was continued for another 125 generations. After 45 generations of exposure at this concentration it was observed that several of treatment replicates were losing viability. To rescue these populations, all replicates were combined. Five new replicates were created by sampling from the mixed pool. These were propagated without selection for AgNP resistance for an additional 40 generations before the new replicates were again exposed to another 80 generations of 125 μg/L (125,000 ng/L) AgNPs.

**Figure 1:**
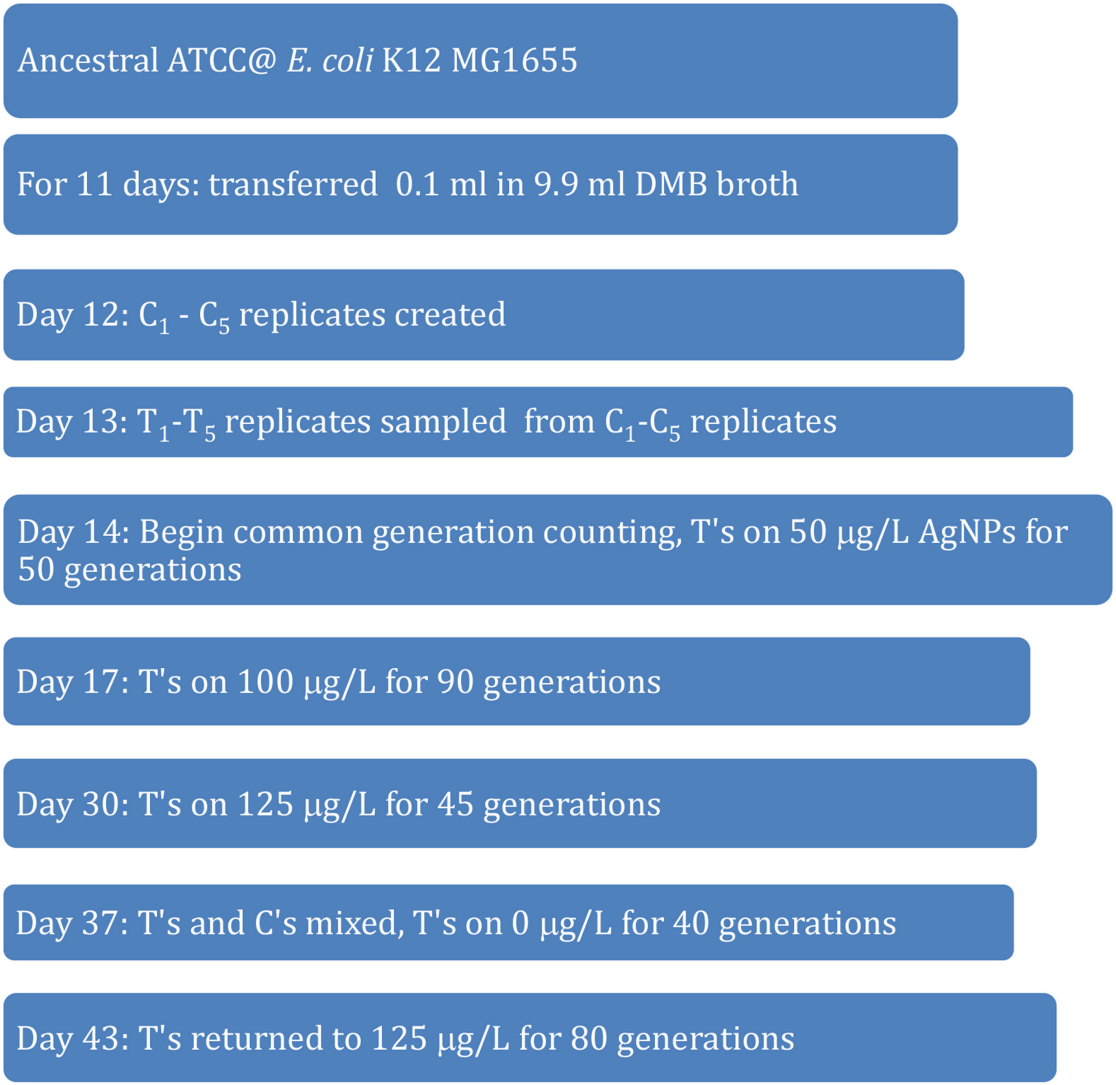
**Evolutionary history of control and treatment populations used in this study**.

After 45 generations of growth in 125 μg/L (125,000 ng/L) AgNPs, these replicates were combined to form a “cocktail” to assay their silver resistance relative to a similarly combined cocktail of control populations. The cocktail protocol was used due to a shortage of labor and materials. Figure 1 shows the schedule of exposure concentrations used in the selection protocol. After 140 generations of culture the exposure for the AgNP selected cultures had increased by 250%.

### Measuring bacterial growth

Bacterial growth in BHI broth samples was assessed by measuring turbidity at 620 nm for hours 0, 3, 6, 12, and 24, using a 98-well plate Synergic Mx spectrophotometer (Biotek, VA USA) using clear polyester 98-well plates.

### Bacterial enumeration

Bacterial populations were determined by spread plating on Davis Minimal Agar (DMA, Sigma Aldrich). In this procedure, samples were withdrawn from inoculated samples at 0 and 24 h and were serially diluted in 0.1% peptone water. Appropriate dilutions were plated (200 μl) onto duplicate DMA plates and colonies were counted after incubation at 37°C overnight.

### Minimum inhibitory concentration (MIC)

MIC (Minimum Inhibitory concentration) is often defined in different terms; however we used “lowest concentration of a particular substance needed to inhibit the growth of a certain population of bacteria” (Kedziora et al., 2013). In this study we used MIC to mean the concentration that inhibited any visible growth of the organism over 24 h. MICs were determined via serial dilution.

### Genomic analysis

Whole genome resequencing was utilized to identify genomic variants associated with the greater AgNP and AgNO_3_ resistance of the treatment populations compared to the controls. DNA was extracted from each replicate population at generation 100 and generation 200 that had been stored in the −80°C freezer (C_1_—C_5_; T_1_—T_5_) as well as from the ancestral *E. coli* K-12 MG1655 stock obtained from ATCC (designated C_0_) for the genomic studies. The cells used for DNA extraction were cultured for 24 h in standard DM broth (without AgNPs). Cell density in these cultures was ~10^8^ cells/ml. Extraction of DNA followed the protocol from Nucleic Acid Sample Preparation for Downstream Analysis: Principles & Methods (GE Health Life Sciences, 2014). The DNA samples were sequenced via the Illumina MiSeq sequencing platform utilizing the Illumina Nextera XT kit for genomic library preparation.

Prior to sequence alignment and variant calling, the Illumina adapter sequences were removed from the MiSeq reads via Partek (http://www.partek.com/). Sequence alignment and variant calling from the generation 100 and 200 samples was achieved by use of the ***breseq*** 0.24rc6 pipeline (Barrick et al., 2009; Deatherage and Barrick, 2014). The ***breseq*** program identifies mutations relative to a reference genome sequence. It is capable of identifying genomic variants including single nucleotide polymorphisms (SNPs), insertion-deletion polymorphisms (indels), and mutations caused by transposable insertion sequence (IS) elements.

## Results

### Phenotypes

**Figure 2:**
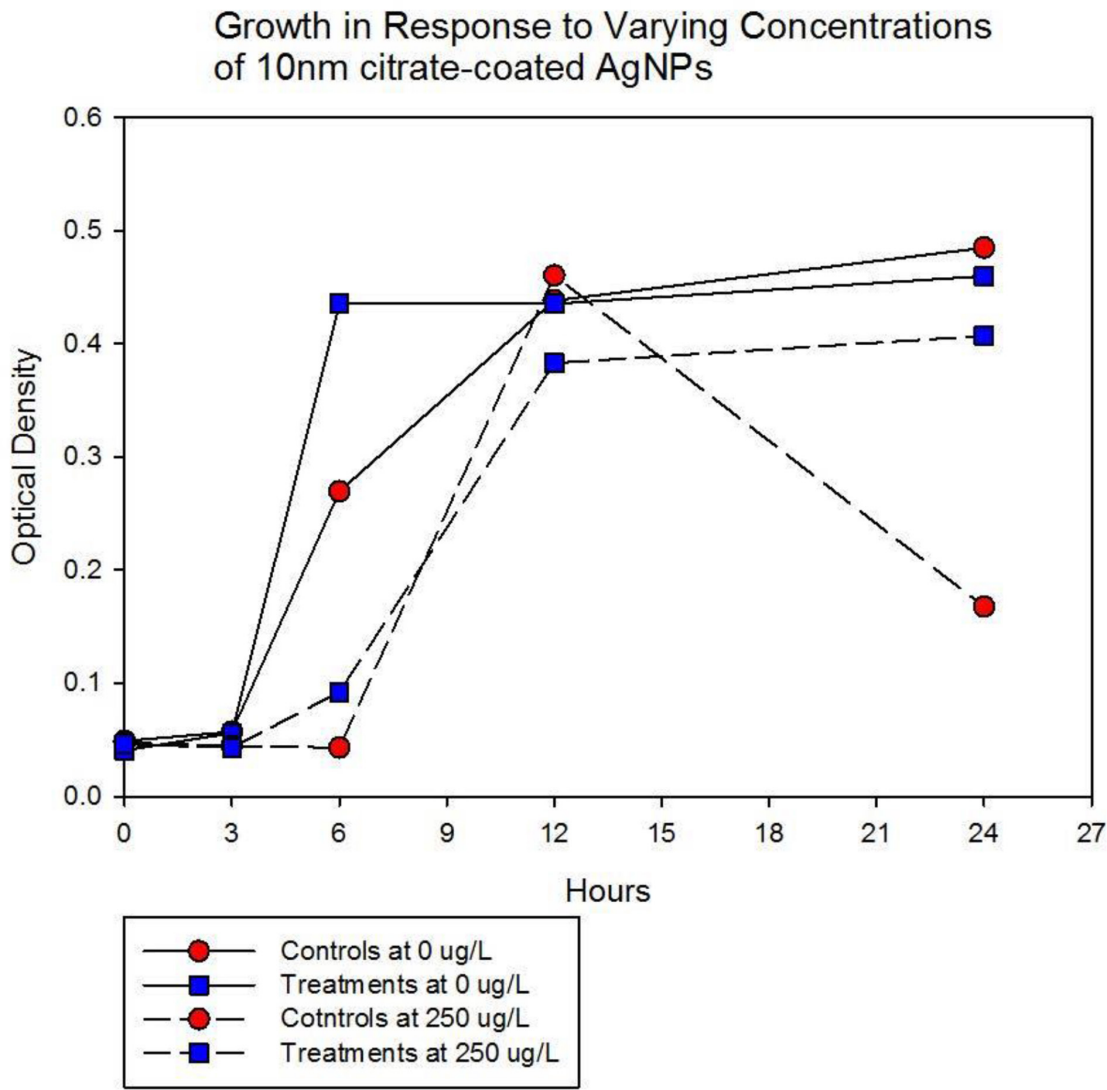
**Optical density measurement of population growth is shown for controls (red circles) and treatment (blue squares) cocktail at generation 162 for 0 μg/L (solid line) and 250 μg/L (250,000 ng/L, dashed line) citrate-coated AgNPs.** Controls show slightly more rapid growth in the absence of AgNPs, at 250 μg/L controls show no growth and treatment growth is delayed by 12 h before exponential growth begins. Neither group showed any growth at higher concentrations (500, 1000 μg/L; 500,000 ng/L, 1,000,000 ng/L.).

Population growth of the control (C_1_—C_5_) and treatment (T_1_—T_5_) populations were measured at generation 162 and 250 in the presence of 10 nm citrate-coated AgNPs by cocktailing the control and treatment replicates. Figure 2 shows the optical density measurement of population growth for control (red circles) and treatment (blue squares) cocktails at generation 162 for 0 μg/L (solid line) and 250 μg/L (250,000 ng/L, dashed line) citrate-coated AgNPs. Controls showed slightly more rapid growth in the absence of AgNPs, and at 250 μg/L controls showed no growth. At 250 μg/L the treatment cocktail growth was delayed by 12 h before growth began. The treatments were able to show approximately a 100-fold increase through growth to saturation during the final 12 h. Neither group showed any growth at higher concentrations (500, 1000 μg/L; 500,000 ng/L, 1,000,000 ng/L.) Figures 3A,B show optical density (OD) measured growth from generation 250 for a cocktail of control and treatment bacteria at the following concentrations of AgNPs (0, 250, 500, and 750 μg/L). In Figure 3A the optical density growth (0.04—0.50) of the controls and treatment populations in 0 μg/L AgNPs corresponded to a 100-fold population increase. This was determined by counting colony-forming units (CFUs) for the cocktails without AgNPs over that same time period. At 250 μg/L both populations show a lag in growth (exponential phases starting at ~6 h) and at 24 h the treatment populations are showing superior growth relative to the control cocktail that shows a decrease in OD from hour 12 to 24. Figure 3B demonstrates that there is no detectable growth of the control cocktail at both concentrations of 500 and 750 μg/L (750,000 ng/L) AgNPs. Conversely, growth is observed in the treatment cocktail, which at 500 μg/L shows an exponential phase after 6 h, but is depressed in growth relative to the absence of AgNPs in the medium (Figure 3A). At 750 μg/L AgNPs the treatment cocktail shows an exponential phase after 12 h and at 24 h has reached a concentration similar to that observed in 250 μg/L.

**Figure 3A:**
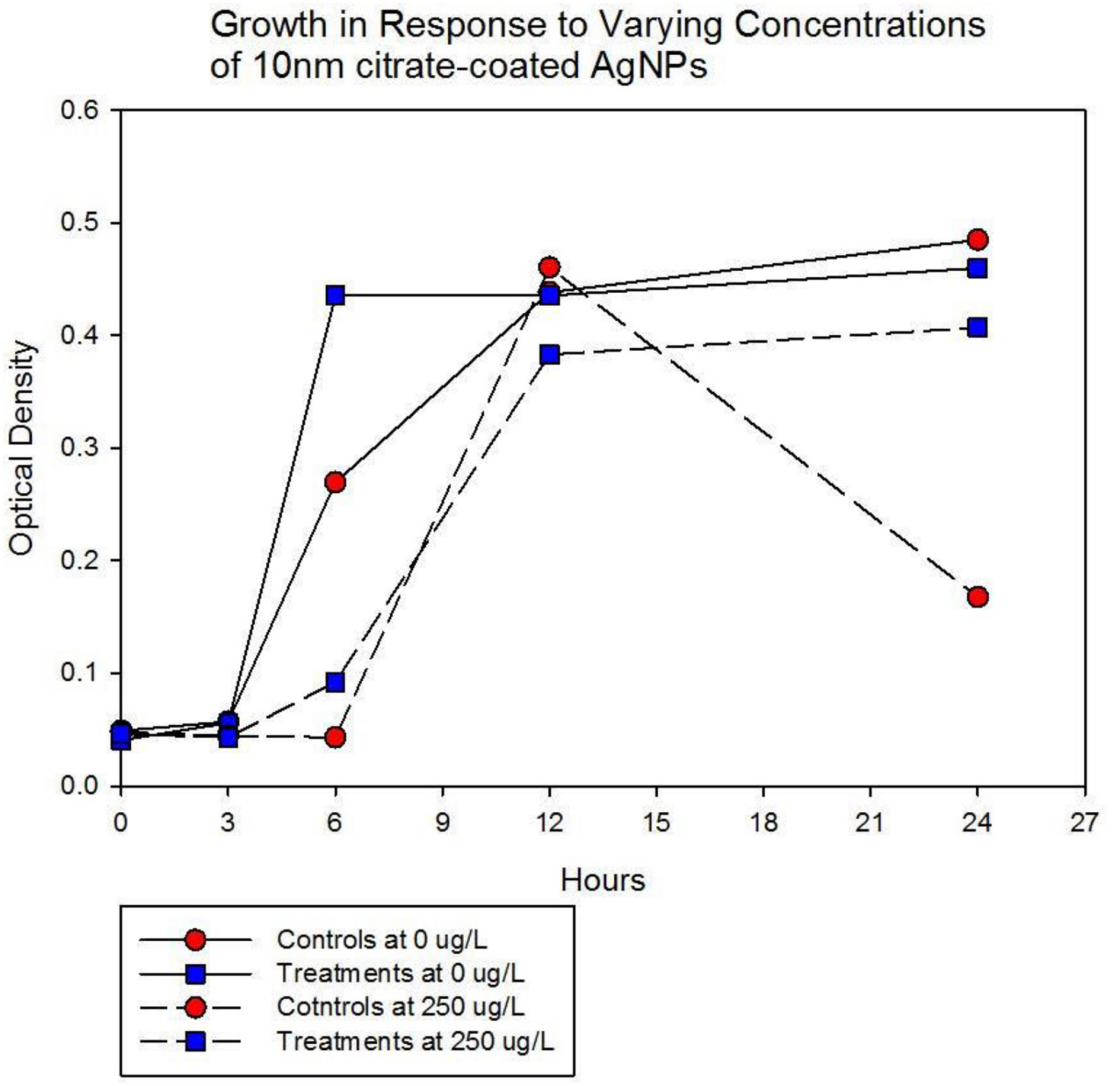
**Population growth over 24 h is shown for control (red circles) and treatment (blue squares) cocktails in response to varying concentrations of 10 nm citrate-coated NPs at generation 250.** The growth of the controls and treatment populations in 0 μg/L AgNPs corresponded to 2 log_10_ population increase. At 250 μg/L both populations show a lag in growth (exponential phases starting at ~6 h) and at 24 h the treatment populations are showing superior growth relative to the control (that shows decrease from hour 12 to 24.).

**Figure 3B:**
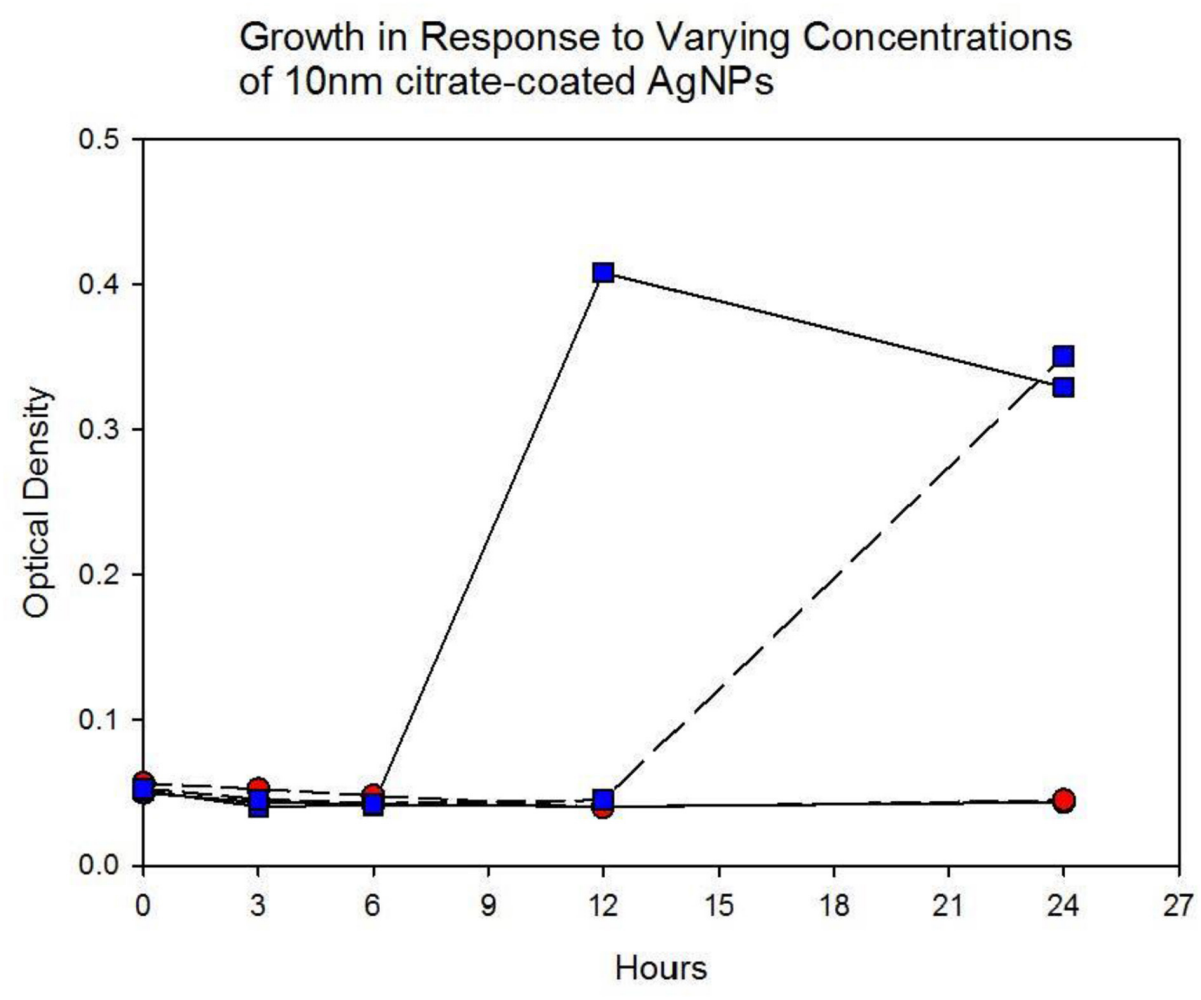
**Population growth over 24 h is shown for control (red circles) and treatment (blue squares) cocktails in response to varying concentrations of 10 nm citrate-coated NPs at generation 250.** There is no detectable growth of the control cocktail at both concentrations of 500 and 750 μg/L (500,000 ng/L and 750,000 ng/L) AgNPs. At 500 μg/L the treatment cocktail does not show an exponential growth phase until after 6 h. At 750 μg/L AgNPs the treatment cocktail does now show an exponential phase until after 12 h and at 24 h has reached an optical density of 0.360 (~>1.5 log_10_ increase).

Figure 4A shows the growth of the control and treatment cocktails in response to AgNO_3_ (ionic silver). Both cocktail populations in 0 μg/L AgNO_3_ exhibit a 2 log_10_ population increase. At 250 μg/L the treatment cocktail does not show an growth phase until after 3 h, but grows to almost a 2log_10_ increase. At this concentration control cocktail does not show an exponential phase until after 6 h and only shows a 1log_10_ increase. Figure 4B shows population growth for control and treatment cocktails in response to 500 and 1000 μg/L of AgNO_3_. There is no detectable growth of the control cocktail at both concentrations of 500 and 1000 μg/L. At 500 μg/L the treatment cocktail does not show an exponential phase until after 12 h, but is depressed in growth relative to the absence of AgNO_3_ in the medium (Figure 4A). At 1000 μg/L AgNO_3_ the treatment cocktail does not show growth until after 12 h and at 24 h shows about a 10-fold increase in cell density.

**Figure 4A:**
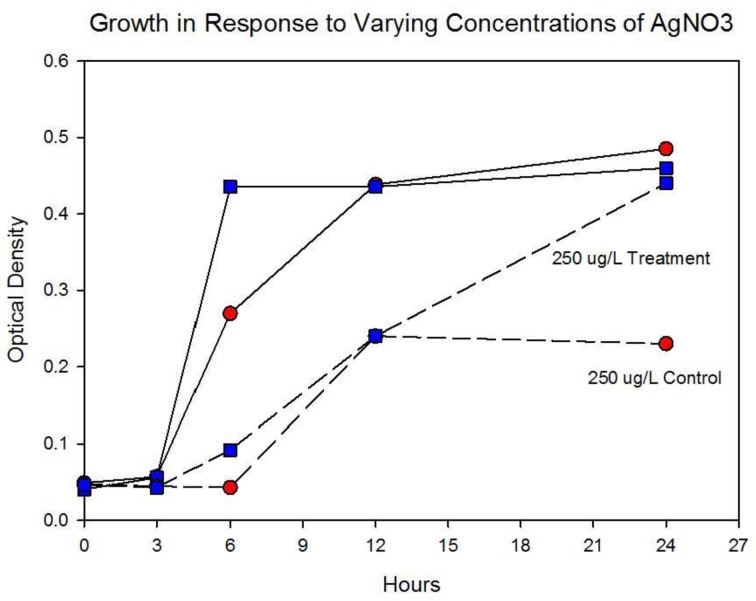
**Population growth over 24 h is shown for control and treatment cocktails in response to varying concentrations of AgNO_3_ at generation 250.** The growth of the controls and treatment populations in 0 μg/L AgNO_3_ corresponded to 2 log_10_ population increase. At 250 μg/L the treatment cocktail does not show an exponential phase until after 3 h, but grows to almost a 2log_10_ increase. At this concentration control cocktail does not show an exponential phase until after 6 h and only shows a 1log_10_ increase.

**Figure 4B:**
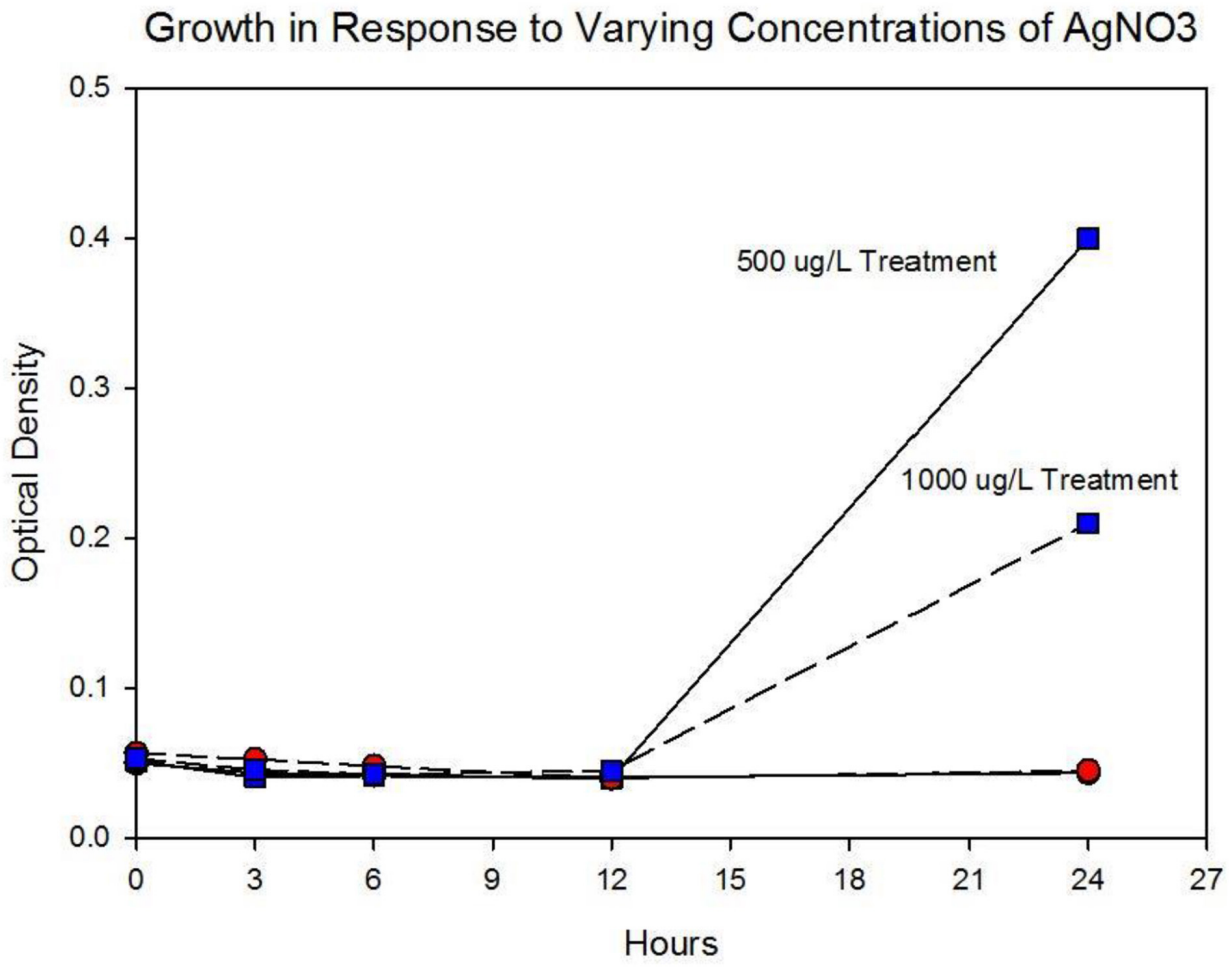
**Population growth over 24 h is shown for control and treatment cocktails in response to varying concentrations of AgNO_3_ at generation 250.** There is no detectable growth of the control cocktail at both concentrations of 500 and 1000 μg/L. At 500 μg/L the treatment cocktail does not show an exponential phase until after 12 h, but is depressed in growth relative to the absence of AgNO_3_ in the medium (Figure 3A). At 1000 μg/L (1,000,000 ng/L) AgNO_3_ the treatment cocktail does now show an exponential phase until after 12 h and at 24 h is about 1log_10_ increase.

Figure 5A shows population growth measured by colony forming units over 24 h for control and treatment cocktails in response to varying concentrations of 10 nm AgNPs at generation 250. Colony forming unit (CFU) measurement is considered a more accurate estimate of bacterial population size compared to optical density (OD). The Figure shows that the CFU and OD results agree. Both cocktails increased by 2.4log_10_ in the absence of AgNPs. There is no detectable difference in growth of the control and treatment cocktails at 100 μg/L. However at 250, 500, and 1000 μg/L the treatment cocktail shows superior growth. At 500 μg/L the treatment cocktail is still growing while the control has shown −1.9 log_10_ decrease, at 1000 μg/L both have decreased with the treatment cocktail showing less reduction. Figure 5B shows population growth measured by colony forming units over 24 h for control and treatment cocktails in response to varying concentrations of AgNO_3_ at generation 250. Both cocktails increased by 2.4 log_10_ in the absence of AgNPs. The data indicate that AgNO_3_ is more effective in reducing bacterial growth compared to the 10 nm AgNPs. This is illustrated by the fact that while the treatment shows positive growth exceeding the control cocktail by ~2 log_10_ units at 100 μg/; both cocktails show decrease at 250, 500, and 1000 μg/L with the treatment cocktail showing less reduction at all concentrations.

**Figure 5A:**
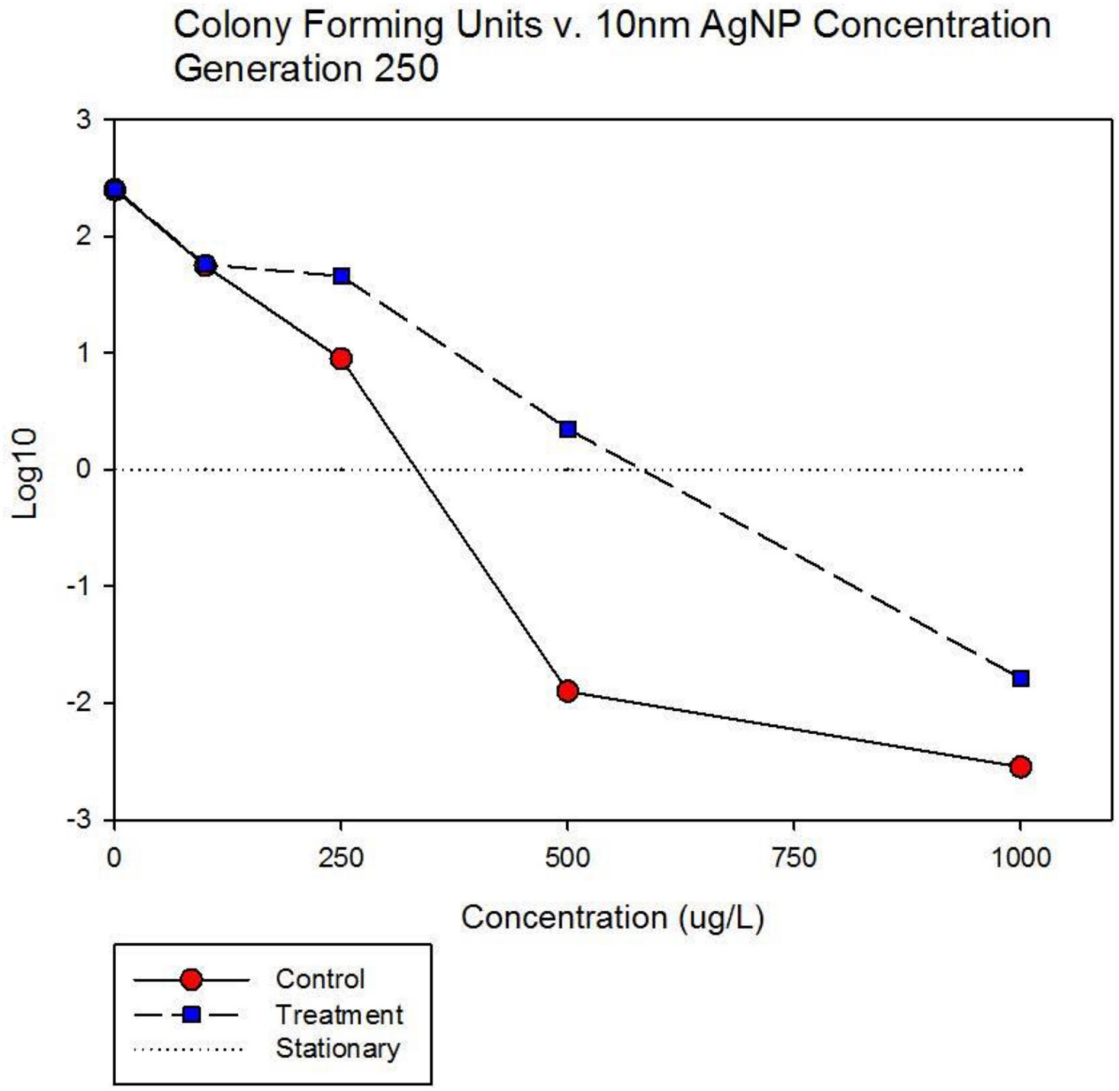
**Population growth measured by colony forming units over 24 h is shown for control and treatment cocktails in response to varying concentrations of 10 nm AgNPs at generation 250.** Both cocktails increased by 2.4 log_10_ in the absence of AgNPs. There is no detectable difference in growth of the control and treatment cocktails at 100 μg/L. However at 250, 500, and 1000 μg/L the treatment cocktail shows superior growth. At 500 μg/L the treatment cocktail is still growing while the control has shown −1.9 log_10_ decrease, at 1000 μg/L both have decreased with the treatment cocktail showing less reduction.

**Figure 5B:**
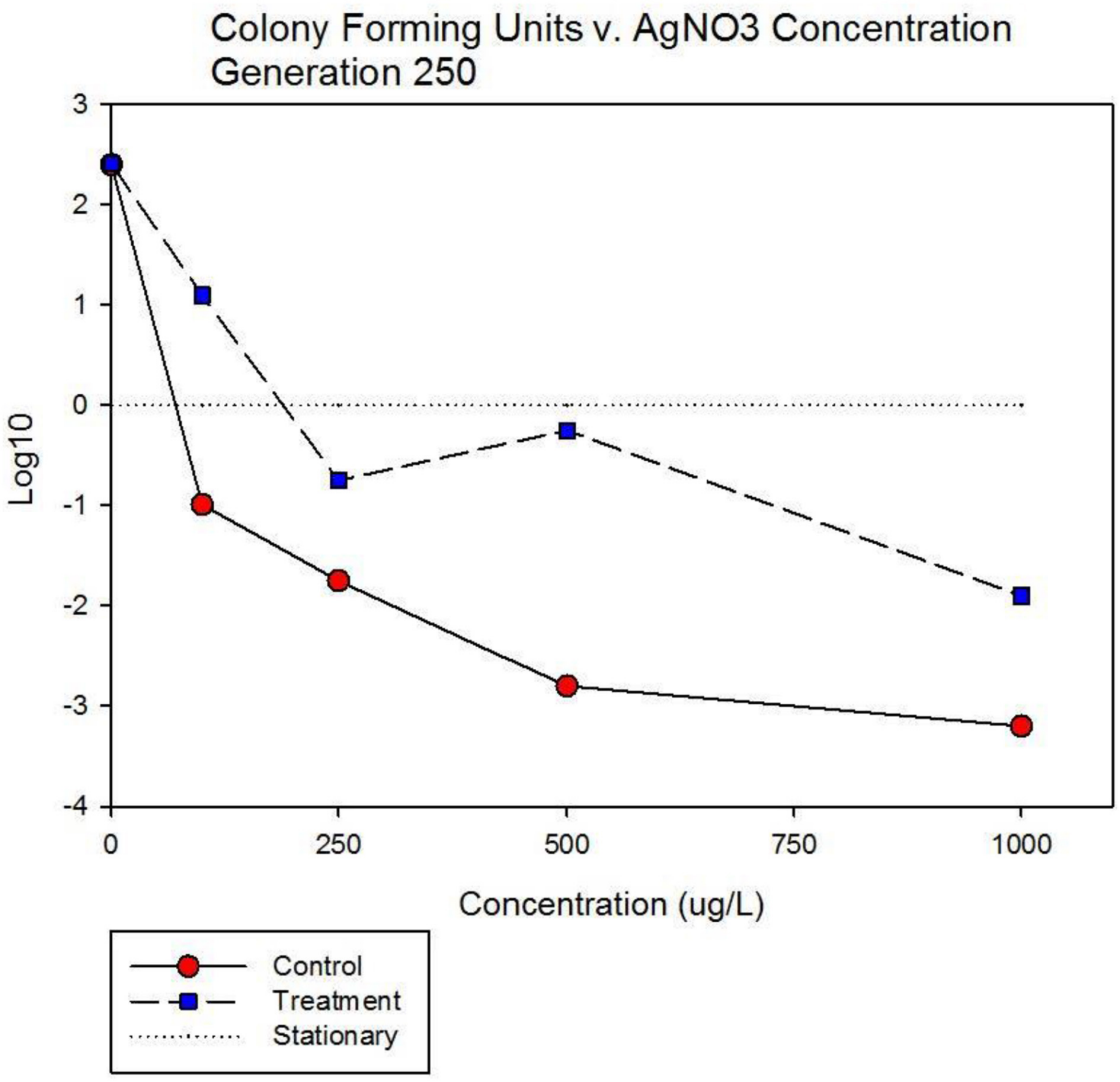
**Population growth measured by colony forming units over 24 h is shown for control and treatment cocktails in response to varying concentrations of AgNO_3_ at generation 250.** Both cocktails increased by 2.4 log_10_ in the absence of AgNO_3_. The treatment shows positive growth exceeding the control cocktail by ~2 log_10_ units at 100 μg/L. However both cocktails show decrease at 250, 500, and 1000 μg/L with the treatment cocktail showing less reduction at all concentrations.

The data from both optical density (OD) and colony forming units (CFU) taken together indicate that by generation 250 of selection (~38.5 days) the treatment populations had evolved resistance to both citrate-coated 10 nm AgNPs and AgNO_3_ (ionic silver). Resistance to 10 nm PVP-coated AgNPs, 40 nm citrate and PVP-coated AgNPs was also illustrated by generation 250 (data not shown).

### Genomics

Tables 2A,B show the genomic variants there were found in the *E. coli* K-12 MG1655 sample received from ATCC, relative to the published K-12 MG1655 genome via breseq. This pipeline uses three types of evidence to predict mutations, read alignments (RA), missing coverage (MC), and new junctions (JC) (Deatherage and Barrick, 2014). Any reads that indicate a difference between the sample and the reference genome that cannot be resolved to describe precise genetic changes are listed as “unassigned.” The unassigned results are not reported in this paper. The variants can be called by either consensus or polymorphism mode. In the former, only variants that are consistent with being present in 100% of the reads in a sample are predicted, and in the latter, variants present at intermediate frequencies are reported. The data in Table 2 are reported in polymorphism mode. These indicate that there are a few differences between the ancestral sample of *E. coli* MG1655 and the reference genome that have been previously documented (Nahku et al., 2011). Most notable is a 776 bp deletion that appears in the *crl* gene at position 257,908. This deletion was fixed (frequency = 1.00) in all reads, as well as mutations at 2,173,361 (Δ2 bp), 3,560,455 (i1bp, G), and 4,296,380 (i2bp, CG). The description of the genes in which each mutation occurs are found in Table 2B.

**Table 2A:** **Ancestral K-12 MG1655 Strain (Polymorphisms) summary of predicted mutations**.

**Position**	**Mutation**	**Freq.**	**Gene**
257,908	Δ776 bp	1.000	*[crl]*
580,302	C→T	0.222	*ylcI ←*
2,173,361	Δ2 bp	1.000	*gatC ←*
2,815,694	A→T	0.182	*gshA ←*
3,271,022	Δ3 bp	0.075	*garK ←*
3,560,455	I + G	1.000	*glpR ←/← glpR*
4,296,060	C→T	0.333	*gltP →/← yjcO*
4,296,380	I + CG	1.000	*gltP →/← yjcO*

All mutations were called via breseq version 0.24rc6. In the mutation column, D, deletion; I, insertion; arrows indicate point mutations. In the gene column, arrows indicate the direction of the gene. If one gene name is shown the mutation is within that gene. If two gene names are shown separated by a slash, then the mutation falls within the intergenic region between those two genes.

**Table 2B:** **Descriptions of predicted mutations in K-12 MG1655 Ancestral Strain**.

**Gene**	**Annotation**	**Description**
[crl]		[crl]
ylcI ←	R48R (AGG→AGA)	DUF3950 family protein, DLP12 prophage
gatC ←	pseudogene (1-2/442 nt)	Pseudogene, galactitol-specific enzyme IIC component of PTS; transport; Transport of small molecules: Carbohydrates, organic acids, alcohols; PTS system galactitol-specific enzyme IIC
gshA ←	L249H (CTT→CAT)	Glutamate-cysteine ligase
garK ←	coding (747-749/1146 nt)	Glycerate kinase I
glpR ←/← glpR	intergenic (−2/+1)	Pseudogene, DNA-binding transcriptional repressor;regulator; Energy metabolism, carbon: Anaerobic respiration; repressor of the glp operon/pseudogene, DNA-binding transcriptional repressor; regulator; Energy metabolism, carbon: Anaerobic respiration; repressor of the glp operon
gltP →/← yjcO	intergenic (+266/+376)	Glutamate/aspartate:proton symporter/Sel1 family TPR-like repeat protein
gltP →/← yjcO	intergenic (+586/+56)	Glutamate/aspartate:proton symporter/Sel1 family TPR-like repeat protein

All mutations were called via breseq version 0.24rc6.

Table 3A shows the mean and SD for selected mutation frequencies for the treatment and control replicates (C_1_—C_5_ and T_1_—T_5_) from generation 100. These replicates were created from a common population that was grown for 11 days in standard DMB media before being established as separate populations. The control populations displayed a total of 28 putative polymorphisms, of which 5 exceeded a frequency of 0.05. The treatment populations displayed 13 polymorphisms, of which 3 exceeded a frequency of 0.05. The controls and treatments shared one SNP at 4,296,060 that was observed in the ancestral K-12 MG1655 strain. This SNP was reduced in frequency and ancestral SNPs 508,302 and 2,815,694 were lost in both groups. The treatment (T_4_) population was the only population fixed (frequency = 1.000) for a mutation in the *cusS* gene (593,497, T—G, D435A, GAC—GCC) and *rpoB* gene (4,182,820, C—T, H526Y, CAC—TAC). The *cusS* gene is the histidine kinase in a two-component regulatory system with *cusR* that senses copper ions. The *rpoB* gene is the RNA polymerase beta subunit. The control (C_4_) population also displayed the *rpoB* mutation (*f* = 0.247). Both the control (C_4_, *f* = 0.223; C_5_ *f* = 1.000) and treatment (T_1_, *f* = 1.000; T_2_, *f* = 1.000; T_3_, *f* = 0.580; T_4_, *f* = 0.000; T_5_, *f* = 1.000) populations shared another mutation in *rpoB* (4,182,803, C—A, P520Q, CCG—CAG). Table 3B gives the annotation and description for selected polymorphic SNPs in generation 100.

**Table 3A:**
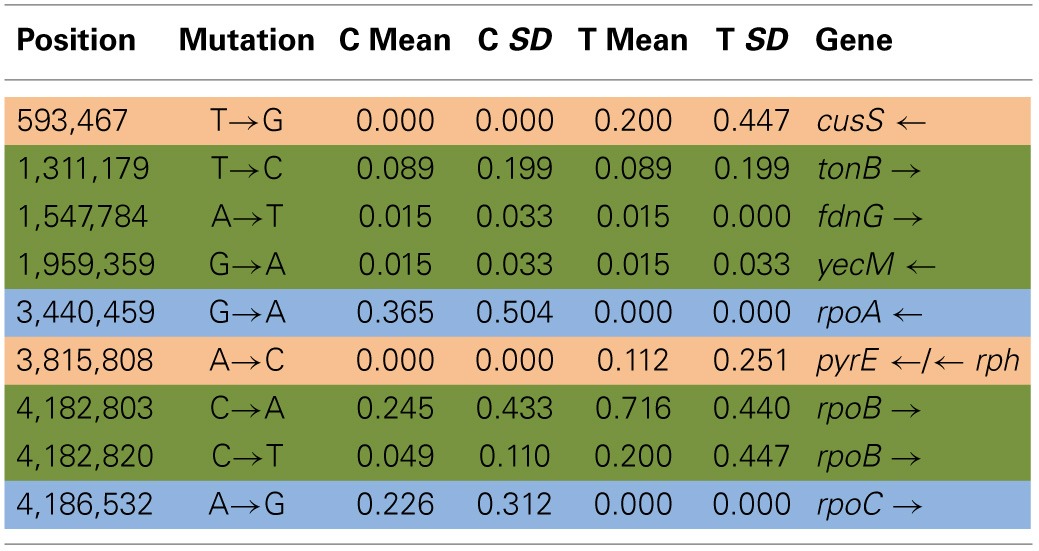
**Control and treatment single nucleotide polymorphisms (SNPs) summary of predicted mutations from generation 100**.

Mean and SD for selected mutation frequencies are shown for the treatment and control replicates (C_1_—C_5_ and T_1_—T_5_) from generation 100. The control populations displayed a total of 28 polymorphisms, of which 5 exceeded a frequency of 0.05. The treatment populations displayed 13 polymorphisms, of which 3 exceeded a frequency of 0.05. Blue-shaded cells, control adaptation; orange-shaded cells, AgNP resistant adaptation; green-shaded cells are adaptations shared by both treatments, but not observed in the ancestral MG1655.

**Table 3B:**
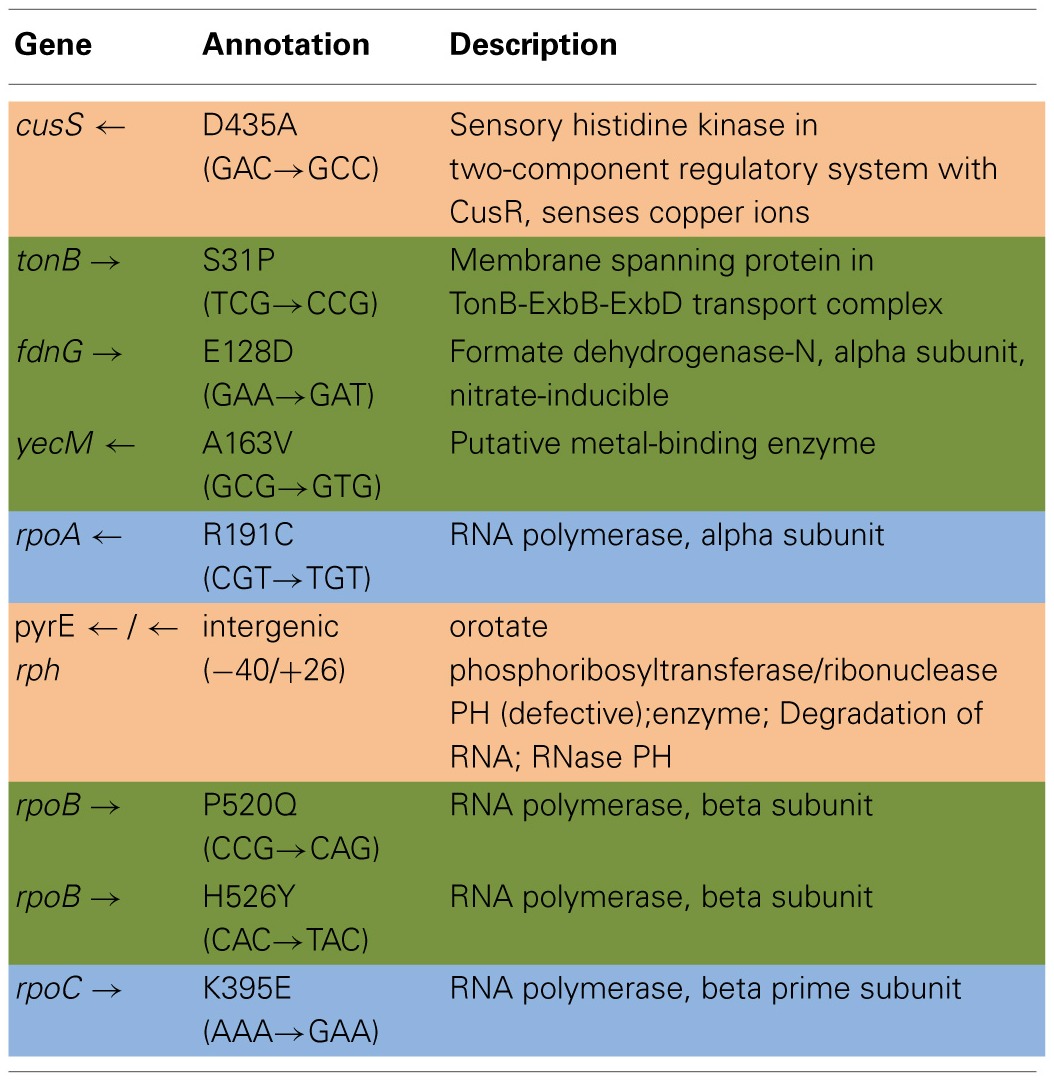
**Description of control and treatment single nucleotide polymorphisms (SNPs) genes from generation 100**.

Blue-shaded cells, control adaptation; orange-shaded cells, AgNP resistant adaptation; green-shaded cells are adaptations shared by both treatments, but not observed in the ancestral MG1655.

Table 4A shows the mean and SD for selected indel frequencies for the treatment and control replicates (C_1_—C_5_ and T_1_—T_5_) from generation 100. The control populations displayed a total of 41 putative polymorphisms, of which 10 exceeded a frequency of 0.05, of these 4 were ancestral and 4 shared with the treatment populations but not ancestral. The treatment populations displayed 45 polymorphisms, of which 10 exceeded a frequency of 0.05, of which 4 were ancestral and 4 were shared with the controls but not ancestral. The ancestral 3 bp deletion at position 3,271,022 was lost in both groups. In addition, the 2 bp deletion at 2,173,361 (*gatC*) seems to be scored by breseq as either a 1 bp or 2 bp deletion. Two additional deletions and 1 insertion appear in both groups (positions 1,299,499; Δ1,999 bp; 1,978,503; Δ776 bp; and 1,979,486; showing an insertion element with an 4 bps duplicated at the target site (IS5(+) +4bp). Table 4B gives the annotation and description for selected polymorphic indels in generation 100.

**Table 4A:**
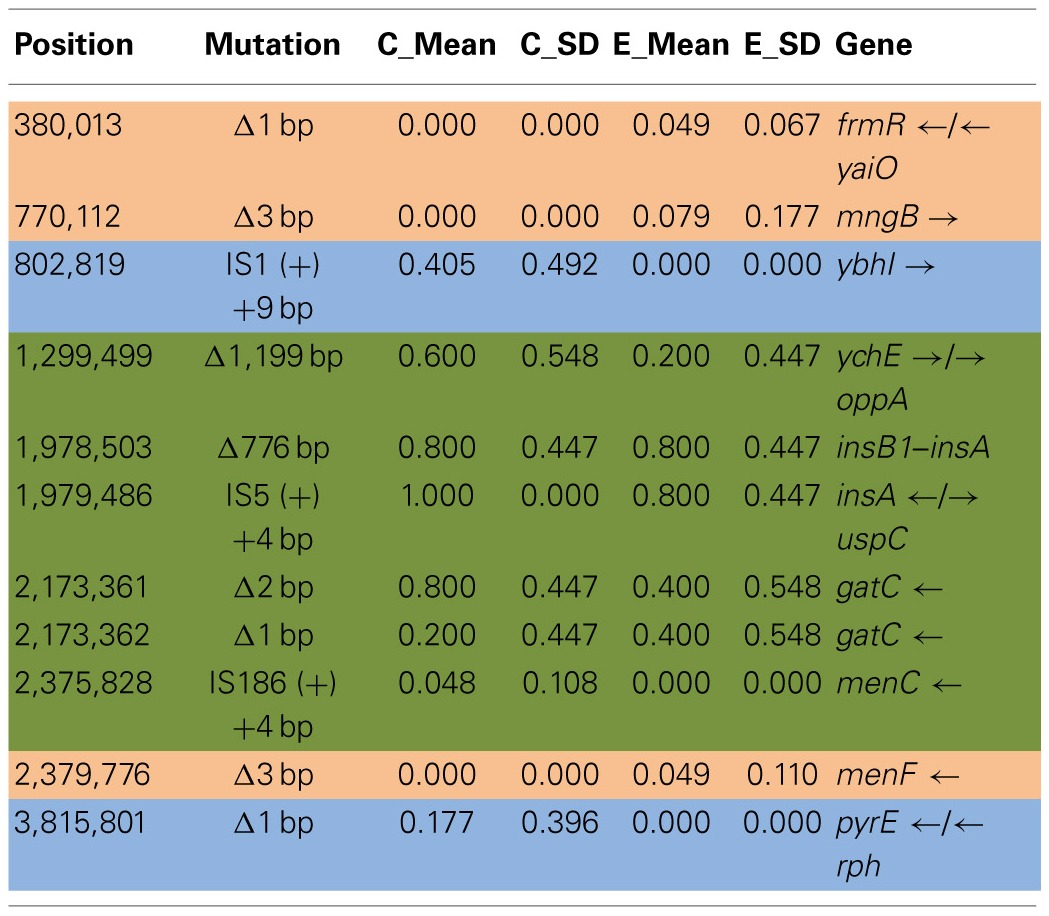
**Control and treatment structural variation from generation 100**.

Mean and SD for selected indel frequencies are shown for the treatment and control replicates (C_1_—C_5_ and T_1_—T_5_) from generation 100. The control populations displayed a total of 41 polymorphisms, of which 10 exceeded a frequency of 0.05, of these 3 were ancestral and five shared with the treatment populations but not ancestral. The treatment populations displayed 45 polymorphisms, of which 10 exceeded a frequency of 0.05, of which 3 were ancestral and 5 were shared with the controls but not ancestral. Blue-shaded cells, control adaptation; orange-shaded cells, AgNP resistant adaptation; green-shaded cells are adaptations shared by both treatments, but not observed in the ancestral MG1655.

**Table 4B:**
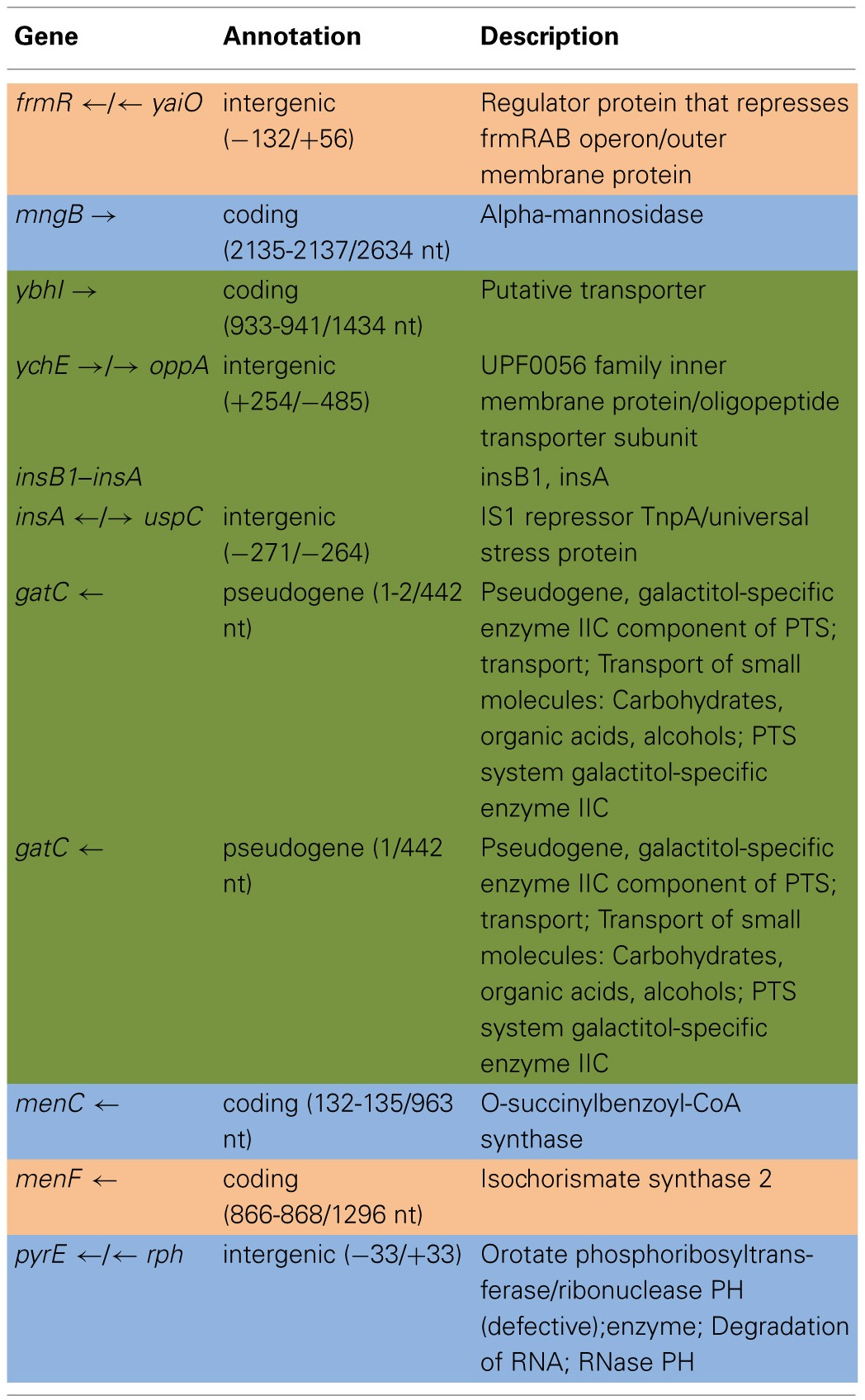
**Description of control and treatment indels from generation 100**.

Blue-shaded cells, control adaptation; orange-shaded cells, AgNP resistant adaptation; green cells are adaptations shared by both treatments, but not observed in the ancestral MG1655.

Table 5A shows the mean and SD for selected mutation frequencies for the treatment and control replicates (C_1_—C_5_ and T_1_—T_5_) from generation 200. The control populations displayed a total of 86 putative polymorphisms, of which 27 exceeded a frequency of 0.05. The treatment populations displayed 16 polymorphisms, of which 8 exceeded a frequency of 0.05. Unlike the data from generation 100 there are no shared SNPs that were not present in the ancestral population (position 4,296,060 still remains in both.) There were 4 mutations of note in the controls. The C_5_ population showed a mutation in *cusR*, a response regulator with *cusS* (position 594,942, E186K, GAA—AAA, *f* = 0.583). C_2_ was fixed for a synonymous mutation in *ccmE*, a periplasmic heme chaperone (position 2,295,168, V71V, GTG—GTA, *f* = 1.000). There were two *rpoC* (RNA polymerase subunit C) mutations (position 4,186,532, K395E, AAA—GAA, *f* = 0.916 in all control replicates) and (position 4,186,605, H419P, CAC—CCC, *f* = 0.500 in C_1_). Particularly notable is the rise in frequency of mutations in *cusS*, *purL* (phosphoribylsylforml-glycineamide synthetase), and *rpoB* in the treatment populations. The *cusS* mutation at position 543,459 is at *f* = 0.803 in T_1_ and at position 593,467 is at *f* = 1.000 for all five replicates. The purL mutation (position 2,694,130) is fixed (*f* = 1.000) in all but T_3_ and the rpoB mutation (4,182,820) is fixed in all five treatment replicates. These mutations are not found in the control populations. This result is particularly interesting in that the *rpoB* mutation (4,182,820) was rare in the controls in generation 100 at *f* = 0.049. In addition, the *rpoB* mutation at position 4,182,803 that was shared by both groups in generation 100 was not detected in either in generation 200. Table 5B gives the annotation and description for selected polymorphic SNPs in generation 200.

**Table 5A:**
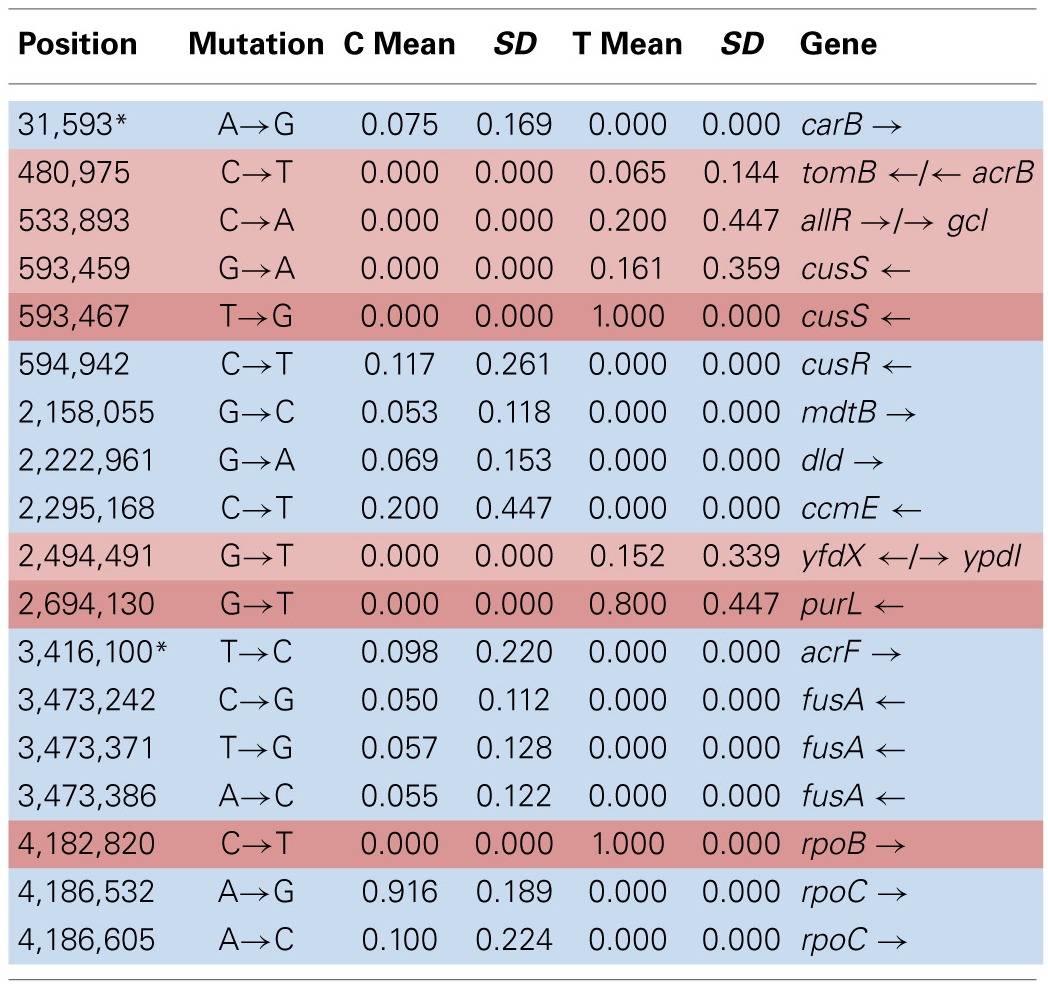
**Control and treatment single nucleotide polymorphisms (SNPs) from generation 200**.

Mean and SD for selected mutation frequencies are shown for the treatment and control replicates (C_1_—C_5_ and T_1_—T_5_) from generation 200. The control populations displayed a total of 86 polymorphisms, of which 27 exceeded a frequency of 0.05. The treatment populations displayed 16 polymorphisms, of which 8 exceeded a frequency of 0.05. Blue-shaded cells, control adaptation; red-shaded cells, AgNP resistant adaptation.

^*^Additional SNPs in carB, 31,599; 602; 621; 644; and 650 ranged from 0.074—0.085 and additional SNPs in acrF were 3,416,107; 118; 121; 124; 136; 148; 151; 158; 161; and 163 ranged from 0.053—0.098.

**Table 5B:**
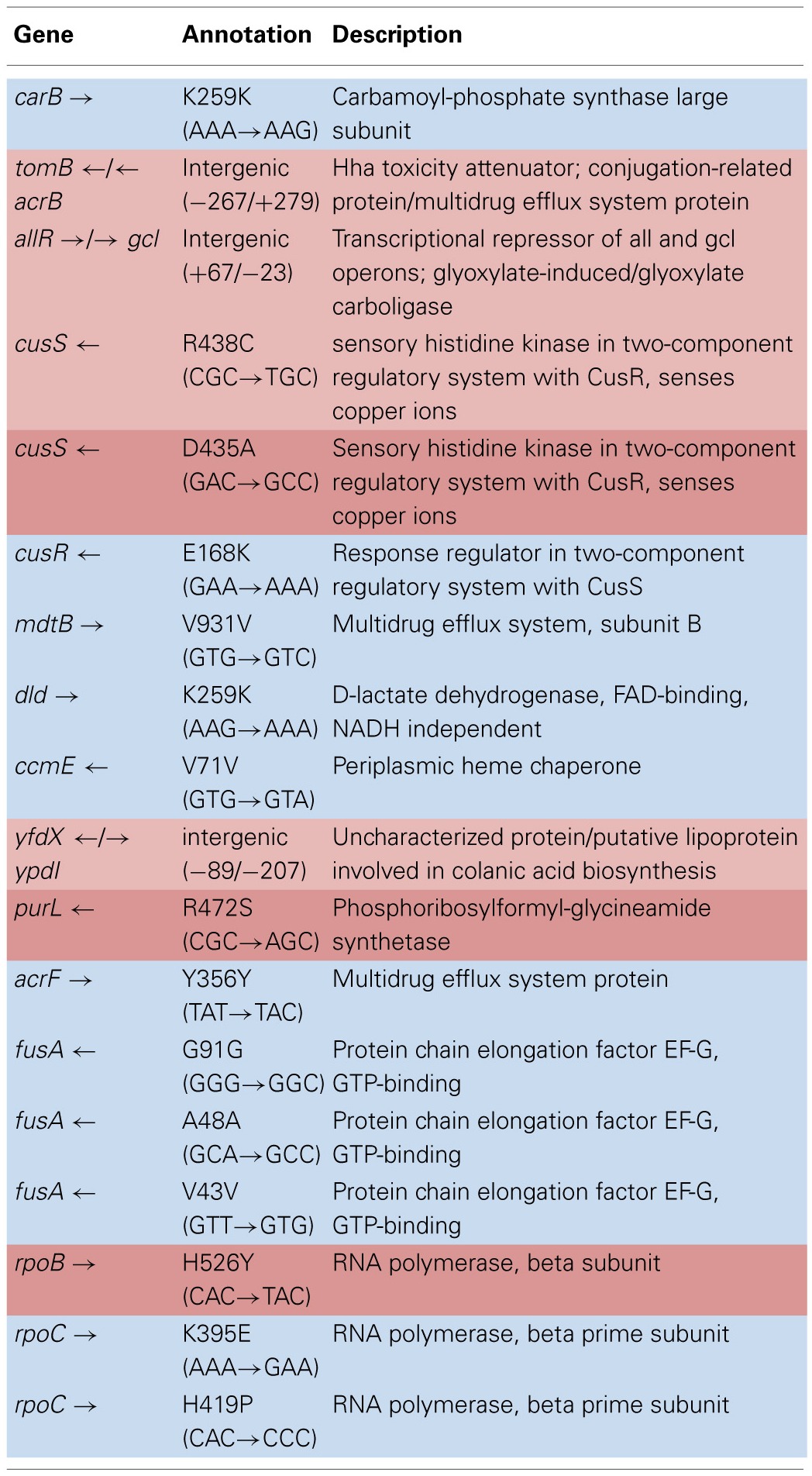
**Description of control and treatment SNPs from generation 100**.

Blue-shaded cells, control adaptation; red-shaded cells, AgNP resistant adaptation.

Table 6A shows the mean and SD for selected indel frequencies for the control and treatment replicates (C_1_—C_5_ and T_1_—T_5_) from generation 200. The control populations displayed a total of 63 putative polymorphisms, of which 9 exceeded a frequency of 0.05, of these 3 were ancestral and 5 were shared with the treatment populations but were not ancestral. The treatment populations displayed 34 polymorphisms, of which 12 exceeded a frequency of 0.05, of which 3 were ancestral and 5 shared with the control populations but not ancestral. The one indel in the control populations was at very low frequency (position 2,552,098, 15-bp insertion in *ypeA*, putative acyl-CoA transferase.). However, the treatment populations had 4 indels of significance (position 2,648,163, Δ7bp, *yfhM*, a periplasmic inner membrane-anchored lipoprotein, T_3_, *f* = 0.498) and three different mutations in *ompR*, response regulator in two-component regulatory system with *envZ*, (position 3,536,264, Δ1bp, T_1_, *f* = 1.000; position 3,536,342, 12bp, T_3_, *f* = 0.947; and position 3,536,570, IS3(−) + 4bp::+TCA, T_5_, *f* = 1.000). Table 6B gives the annotation and description for selected polymorphic indels in generation 200.

**Table 6A:**
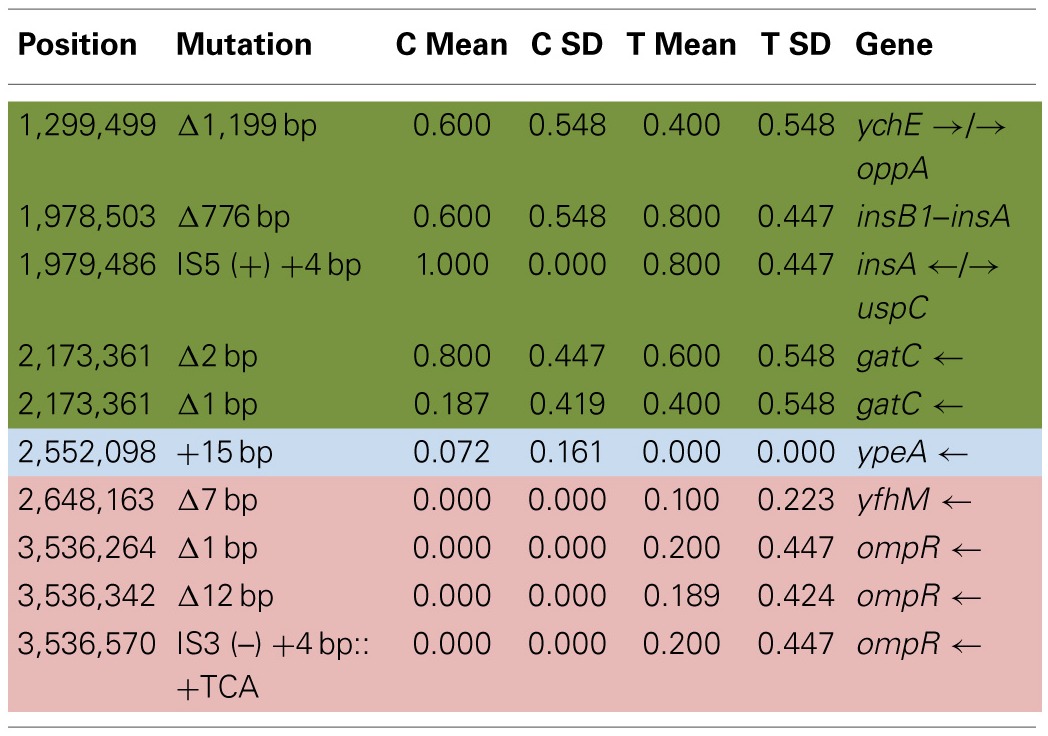
**Control and treatment structural variation from generation 200**.

Mean and SD for selected indel frequencies are shown for the treatment and control replicates (C_1_—C_5_ and T_1_—T_5_) from generation 200. The control populations displayed a total of 63 polymorphisms, of which 9 exceeded a frequency of 0.05, of these 3 were ancestral and 5 were shared with the treatment populations but were not ancestral. The treatment populations displayed 34 polymorphisms, of which 12 exceeded a frequency of 0.05, of which 3 were ancestral and 5 shared with the control populations but not ancestral. Blue-shaded cells, control adaptation; red-shaded cells, AgNP resistant adaptation; green-shaded cells are adaptations shared by both treatments, but not observed in the ancestral MG1655.

**Table 6B:**
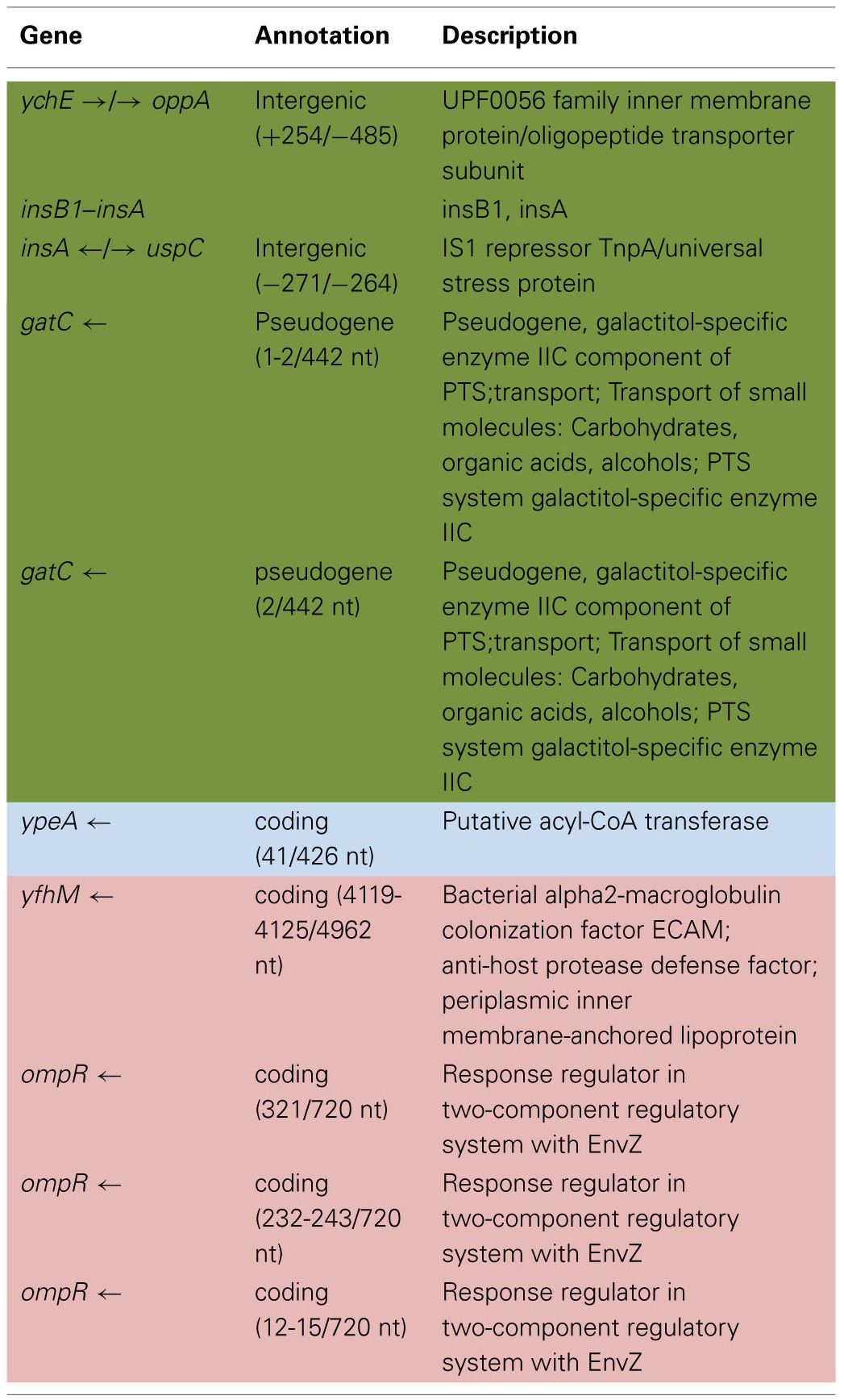
**Description of control and treatment indels from generation 200**.

Blue-shaded cells, control adaptation; red-shaded cells, AgNP resistant adaptation; green-shaded cells are adaptations shared by both treatments, but not observed in the ancestral MG1655.

## Discussion

The purpose of this study was to determine how rapidly and by what kinds of genomic changes AgNP resistance could evolve in a relatively naïve bacterium, *E. coli* K-12 MG1655. Here we have shown this bacterium can rapidly evolve resistance to AgNPs. By generation 162 we observed phenotypic evidence that the AgNP resistance of the treatment populations was greater than that of the controls. By generation 250 we show definite resistance to 10 nm citrate-coated AgNPs as well as to ionic Ag^+^ (AgNO_3_). Subsequent selection actually increased both AgNP and AgNO_3_ treatment population resistance by generation 300 to between 4.66 and 1.40 times the MIC of the controls to various sorts of AgNP, but also to greater than 26 times the AgNO_3_ resistance of the controls (Graves, 2014). The genomic analysis demonstrates that resistance alleles were already accumulating in the AgNP resistant populations by generation 100. The increase in resistance we have observed does not seem to be the result of major genomic changes. Rather, by generation 200, mutations in four genes had swept to fixation (or nearly so) in all or some of the treatment populations. Not surprisingly, a mutation occurred in a bacterial system that is known to play a role in regulating heavy metal concentration in the intercellular environment (*cusS*; D435A (GAC→GCC) a non-synonymous point mutation at position 593,467; this is a sensory histidine kinase from the *ompR* family that is in a two-component regulatory system with CusR, that senses copper ions). This mutation was fixed in all five populations. Data from generation 100 suggests that it originated in T_4_. A second mutation in *cusS* (position 593,459, R438C, CGC—TGC, *f* = 0.803) was also scored in T_1._ (Lok et al., 2008) as well as (Gudipathy and McEvoy, 2014) summarize the role of *cusS* in silver resistance. CusS is part of the two-component sensor/responder system located next to the CusCFBA efflux system in the *E. coli* genome. CusCFBA is analogous to (and shares high sequence homology with) SilCFBA the plasmid-encoded silver resistance mechanism of *Salmonella typhimurium*. CusS is a sensor that is a membrane associated gene histidine kinase. It is autophosphorylated upon external stimuli sensed in the periplasmic domain (in this case, Ag^+^ or Cu^2+^ stress). The *cusS* gene is highly conserved in other *E. coli* strains, as evidenced by BLAST search using the ancestral *E. coli* K-12 MG1655 *cusS* sequence. The search returned *cusS* matches from 134 *E. coli* and *Shigella* strains that had 91–100% identity to the MG1655 sequence.

The second mutation was in *purL* (R472S (CGC→AGC), a non-synonymous point mutation at position 2,694,130; phosphoribosylformyl-glycineamide synthetase). This gene catalyzes conversion of 5′-phosphoribosylformylglycinamide (FGAR) to formylglycinamide in the presence of glutamine and ATP for de novo purine nucleotide biosynthesis (Sampei and Mizobuchi, 1989). BLAST search confirmed that the K-12 MG1655 *purL* consensus sequence was highly conserved across *E. coli* strains, 100 matches showed from 98–100% identity. Again, the AgNP resistant mutation was not found in any of the *E. coli* or *Shigella spp*. sequences uncovered by the search.

The third mutation was in RNA polymerase beta subunit, *rpoB*, (H526Y (CAC→TAC) a non-synonymous point mutation at position 4,182,820; RNA polymerase, beta subunit). It was fixed in all treatment populations, and also was first observed in C_4_ (*f* = 0.223) and T_4_ (*f* = 1.000) in generation 100. By generation 200, C_4_ had lost the mutation and it had become fixed in all treatment populations. Adaptive mutations in RNA polymerase are often found in *E. coli* evolution experiments (Conrad et al., 2011), as altering its activity can have large impacts on global gene expression patterns (Conrad et al., 2010).

The adaptive character of both of the *cusS* and *rpoB* are indicated by the fact that they are non-synonymous mutations that occur within active sites of their enzymes. The *cusS* mutation is found in the histidine-kinase, DNA gyrase B, and HSP90-like ATPase domain of the protein (Li et al., 2008; ecogene^1^). The *rpoB* mutation occurs within the Rpb2 domain 3 site of the RNA polymerase B (Cramer et al., 2001; ecogene^2^). The *purL* mutation occurs prior to the enzyme's active sites (257—389; 432—589; and 822—966; Li et al., 1999; ecogene^3^). In generation 100, the *rpoB* mutation was also at high frequency in C_4_ at *f* = 0.247. This might indicate that this mutation was a general adaptation that was useful in both the non-Ag^+^ and Ag^+^ environments. However, by generation 200 the C_4_ population had lost the *rpoB* mutation, while it had swept to fixation in the treatment populations. Thus it is possible that the benefit that *rpoB* gave was greater for the treatments as compared to the controls. The best way to determine if there is any specific Ag^+^ resistance derived from any of these mutations will be to do allelic exchange experiments that place these mutations into the ancestral bacterium and determine if either individually or together they improve AgNP resistance.

In addition to the point mutations observed in AgNP populations in generation 200, at least three of the indels scored may be of particular significance. Three indels (position 3,536,264, Δ1bp, *ompR*, T_1_, *f* = 1.000; position 3,536,342, Δ12bp, *ompR*, T_2_, *f* = 0.947; position 3,536,570, IS3(-) +4bp::TCA, *ompR*, T_5_, *f* = 1.000) were scored. OmpR is a DNA binding protein that is involved in regulating gene networks (Quinn et al., 2014). While its sequence is highly conserved in gram negative bacteria, the collection of genes that it's governs can vary widely. Rhee et al. (2008) have shown that OmpR protein has only moderate specificity for its DNA sites and that this allows new specificity to evolve via only a few mutational steps. Furthermore, OmpR has the potential to respond to more than one environmental signal. In *Salmonella Typhimurium* it responds to acid stress (Quinn et al., 2014). This bacterium is also of great interest with regards to silver resistance, as silver resistant strains have been isolated from both silver mines and clinical environments (McHugh et al., 1975). Given our results we suggest that these indels in the *ompR* sequence of our AgNP resistant populations may be producing an OmpR protein that plays a new role in regulating responses to Ag^+^ resistance.

### Selective sweeps

The design of this experiment is incapable of addressing whether the increase in AgNP resistance mutations occurred by hard or soft sweeps. We began the experiment by sampling the ancestral ATCC 47076 strain of K-12 MG1655 and growing up a stock culture for 11 days (~71 generations). During this time mutations began to accumulate in the stock culture that were not present in the ancestral strain. The control and treatment replicates were sampled from the stock culture and thus share genetic diversity and recent common ancestry with each other (T_1_ sampled from C_1_, T_2_ sampled from C_2_, etc.). For the first 100 generations of the experiment, each of the replicate populations evolved separately from all others. In the control replicates at generation 52/56 scored SNP/indel mutations (not ancestral) were unique to a specific replicate and most were rare. For the treatment populations, 43/46 scored SNP/indel mutations (not ancestral) were unique to a specific population, however two of interest were already at very high frequency (T_4_, position 597,467, cusS, *f* = 1.000 and in T_1_, T_2_, T_3_, T_5_, position 4,183,803, *rpoB*, *f* = 1.000, 1.000, 0.580, and 1.000; T_4_, position 4,182,820, *rpoB*, *f* = 1.000). The *rpoB* mutations were shared with the controls (C_4_, C_5_, position 4,183,803, *rpoB*, *f* = 0.223, 1.000 and C_3_, position 4,182,820, *rpoB*, *f* = 0.247). Given that the control and treatment populations were derived from a stock population that evolved for 71 generations, it is possible that all of the polymorphisms were established in the stock population before the AgNP resistance selection began.

After generation 141 the treatment populations were cocktailed to rescue the poor preforming replicates. This means that cells from the T_4_ replicate were spread into the other populations. By generation 200, the control populations had 138/143 putative SNP/indel mutations that were unique to a specific population, and the treatment populations had 39/42 SNP/indel putative mutations that were unique to a specific population. The later result is somewhat startling, given the mixing of the treatments populations in generation 141. By generation 200, the *cusS* and *rpoB* mutations (first scored in T_4_) had swept to fixation in all treatment populations, the *purL* mutation was fixed in 4/5 treatment populations (but had not been detected in generation 100). The most significant AgNP resistance associated indels (*ompR*) were also unique to the treatment populations.

### Future directions

We are currently engaged in research to address the evolvability of AgNp (and general Ag^+^ ion resistance) resistance in *E. coli* K-12 MG1655. Our AgNP resistant populations were carried to generation 305. At the time of this writing, sequencing of the generation 300 controls and treatments is underway. We have shown that AgNP and AgNO3 resistance has increased in these populations. Our goal will be to determine if the genomic variants we scored in generation 200, as well as new variants that may have accumulated in the treatment replicates are involved in bringing about this increased resistance. We have also selected 18 new replicate populations for resistance to AgNO_3_. These populations were all founded by single colonies derived from the ancestral K-12 MG1655 and were never mixed. This will allow us to address questions concerning mutational history of individual replicates as well as to determine if evolutionary convergence occurs with regards to AgNO_3_ resistance. We also wish to test whether and which genomic variants scored in the AgNP resistance studies reappear in the AgNO_3_ resistance replicates. Ultimately for definitive proof of the contribution of each mutation we score to AgNP or Ag^+^ resistance we will need to perform allelic exchange experiments. This give us direct evidence that an individual mutation or a set of mutations improve AgNP or Ag^+^ ion resistance in the genetic background of our original *E. coli* K-12 MG1655 bacterium.

## Conclusion

Experimental evolution was able to rapidly evolve resistance to 10 nm citrate-coated AgNPs in a relatively naïve bacterium, *E. coli* K-12 MG1655. Resistance required relatively few mutational steps. Thus, these therapies must contend not only with microbes receiving heavy resistance genes via horizontal transfer but also with AgNP resistance readily arising from *de novo* mutations in existing genes. This outcome does not bode well for the sustained use of AgNPs as “miracle” antimicrobials.

### Conflict of interest statement

The authors declare that the research was conducted in the absence of any commercial or financial relationships that could be construed as a potential conflict of interest.

## Footnotes

^1^http://www.kegg.jp/ssdb-bin/ssdb_motif?kid=eco:b0570.

^2^http://www.kegg.jp/ssdb-bin/ssdb_motif?kid=eco:b3987.

^3^http://www.kegg.jp/ssdb-bin/ssdb_motif?kid=eco:b2557.
